# Supplementary material for: Multi‐dataset identification of innovative feature genes and molecular mechanisms in keratoconus
Source: J Cell Mol Med. 2024 Sep 19;28(18):e70079. doi: 10.1111/jcmm.70079 (PMC11412914; doi:10.1111/jcmm.70079)
Supplement: Supplementary file 2 — Material S2. [file JCMM-28-e70079-s002.zip › 7.GSEA analysis/3.DBNDD1/KEGG_ARRHYTHMOGENIC_RIGHT_VENTRICULAR_CARDIOMYOPATHY_ARVC.html]

Details for gene set KEGG\_ARRHYTHMOGENIC\_RIGHT\_VENTRICULAR\_CARDIOMYOPATHY\_ARVC[GSEA]

|  || Dataset | DBNDD1.group.cls#h\_versus\_l |
| Phenotype | group.cls#h\_versus\_l |
| Upregulated in class | l |
| GeneSet | KEGG\_ARRHYTHMOGENIC\_RIGHT\_VENTRICULAR\_CARDIOMYOPATHY\_ARVC |
| Enrichment Score (ES) | -0.5139755 |
| Normalized Enrichment Score (NES) | -1.5953281 |
| Nominal p-value | 0.013752456 |
| FDR q-value | 1.0 |
| FWER p-Value | 0.622 |
Table: GSEA Results Summary

  

Fig 1: Enrichment plot: KEGG\_ARRHYTHMOGENIC\_RIGHT\_VENTRICULAR\_CARDIOMYOPATHY\_ARVC      
 Profile of the Running ES Score & Positions of GeneSet Members on the Rank Ordered List

  

| SYMBOL | TITLE | RANK IN GENE LIST | RANK METRIC SCORE | RUNNING ES | CORE ENRICHMENT || 1 | CACNB1 | na | 587 | 0.431 | 0.0215 | No |
| 2 | CACNA1F | na | 1766 | 0.316 | 0.0156 | No |
| 3 | CACNG5 | na | 2873 | 0.257 | 0.0065 | No |
| 4 | CACNG7 | na | 4144 | 0.209 | -0.0116 | No |
| 5 | SGCD | na | 4837 | 0.185 | -0.0151 | No |
| 6 | DAG1 | na | 5307 | 0.170 | -0.0135 | No |
| 7 | ITGA4 | na | 7459 | 0.114 | -0.0654 | No |
| 8 | EMD | na | 7789 | 0.108 | -0.0653 | No |
| 9 | CACNG1 | na | 8316 | 0.098 | -0.0717 | No |
| 10 | LAMA2 | na | 9618 | 0.077 | -0.1025 | No |
| 11 | ITGB7 | na | 9817 | 0.074 | -0.1016 | No |
| 12 | CACNA2D3 | na | 10208 | 0.068 | -0.1068 | No |
| 13 | ATP2A2 | na | 11124 | 0.057 | -0.1281 | No |
| 14 | DSC2 | na | 11532 | 0.052 | -0.1352 | No |
| 15 | CTNNA2 | na | 11866 | 0.048 | -0.1405 | No |
| 16 | ACTN4 | na | 12890 | 0.039 | -0.1666 | No |
| 17 | CACNA1D | na | 13311 | 0.036 | -0.1755 | No |
| 18 | DES | na | 13515 | 0.034 | -0.1784 | No |
| 19 | CACNB2 | na | 14012 | 0.030 | -0.1900 | No |
| 20 | DSP | na | 14115 | 0.029 | -0.1904 | No |
| 21 | ACTB | na | 14636 | 0.025 | -0.2031 | No |
| 22 | CACNG8 | na | 15428 | 0.020 | -0.2242 | No |
| 23 | DMD | na | 15967 | 0.016 | -0.2383 | No |
| 24 | CTNNB1 | na | 16378 | 0.013 | -0.2489 | No |
| 25 | ITGA2B | na | 16436 | 0.013 | -0.2494 | No |
| 26 | ACTN2 | na | 17576 | 0.006 | -0.2817 | No |
| 27 | ACTG1 | na | 18138 | 0.002 | -0.2978 | No |
| 28 | CTNNA1 | na | 18276 | 0.001 | -0.3017 | No |
| 29 | JUP | na | 19747 | -0.011 | -0.3431 | No |
| 30 | TCF7L2 | na | 20073 | -0.014 | -0.3513 | No |
| 31 | LMNA | na | 20345 | -0.016 | -0.3577 | No |
| 32 | ITGB4 | na | 20511 | -0.017 | -0.3609 | No |
| 33 | GJA1 | na | 21395 | -0.026 | -0.3841 | No |
| 34 | ITGA9 | na | 21410 | -0.026 | -0.3822 | No |
| 35 | ITGB5 | na | 21935 | -0.031 | -0.3945 | No |
| 36 | CACNA1C | na | 23124 | -0.045 | -0.4248 | No |
| 37 | TCF7 | na | 23571 | -0.051 | -0.4332 | No |
| 38 | ITGA3 | na | 24015 | -0.056 | -0.4409 | No |
| 39 | ITGAV | na | 25782 | -0.083 | -0.4845 | No |
| 40 | ITGA11 | na | 26521 | -0.098 | -0.4970 | No |
| 41 | ITGA2 | na | 26752 | -0.102 | -0.4945 | No |
| 42 | TCF7L1 | na | 26813 | -0.104 | -0.4870 | No |
| 43 | CDH2 | na | 26913 | -0.106 | -0.4805 | No |
| 44 | CTNNA3 | na | 27076 | -0.110 | -0.4754 | No |
| 45 | CACNG4 | na | 28414 | -0.141 | -0.5014 | Yes |
| 46 | ITGA6 | na | 28732 | -0.150 | -0.4973 | Yes |
| 47 | PKP2 | na | 28812 | -0.152 | -0.4860 | Yes |
| 48 | ITGA7 | na | 29075 | -0.159 | -0.4794 | Yes |
| 49 | ITGB1 | na | 29102 | -0.160 | -0.4659 | Yes |
| 50 | SGCA | na | 29145 | -0.161 | -0.4528 | Yes |
| 51 | SGCB | na | 29234 | -0.164 | -0.4408 | Yes |
| 52 | CACNB3 | na | 29250 | -0.164 | -0.4266 | Yes |
| 53 | CACNA2D4 | na | 29251 | -0.164 | -0.4120 | Yes |
| 54 | ITGB3 | na | 29286 | -0.165 | -0.3982 | Yes |
| 55 | ITGB6 | na | 29433 | -0.170 | -0.3873 | Yes |
| 56 | DSG2 | na | 29437 | -0.170 | -0.3723 | Yes |
| 57 | SGCG | na | 29686 | -0.179 | -0.3635 | Yes |
| 58 | ACTN1 | na | 30298 | -0.197 | -0.3636 | Yes |
| 59 | ITGB8 | na | 30390 | -0.200 | -0.3484 | Yes |
| 60 | ITGA1 | na | 31047 | -0.222 | -0.3475 | Yes |
| 61 | CACNG6 | na | 31154 | -0.226 | -0.3304 | Yes |
| 62 | SLC8A1 | na | 32046 | -0.266 | -0.3324 | Yes |
| 63 | RYR2 | na | 32172 | -0.274 | -0.3116 | Yes |
| 64 | ITGA10 | na | 32340 | -0.284 | -0.2912 | Yes |
| 65 | CACNA2D2 | na | 32648 | -0.304 | -0.2730 | Yes |
| 66 | LEF1 | na | 32857 | -0.317 | -0.2507 | Yes |
| 67 | CACNG2 | na | 33176 | -0.343 | -0.2294 | Yes |
| 68 | CACNA2D1 | na | 33832 | -0.408 | -0.2120 | Yes |
| 69 | ACTN3 | na | 33884 | -0.415 | -0.1765 | Yes |
| 70 | CACNB4 | na | 33927 | -0.420 | -0.1404 | Yes |
| 71 | ITGA5 | na | 34082 | -0.440 | -0.1056 | Yes |
| 72 | ITGA8 | na | 34117 | -0.445 | -0.0670 | Yes |
| 73 | CACNA1S | na | 34256 | -0.474 | -0.0287 | Yes |
| 74 | CACNG3 | na | 34262 | -0.475 | 0.0134 | Yes |
Table: GSEA details [plain text format]

  

Fig 2: KEGG\_ARRHYTHMOGENIC\_RIGHT\_VENTRICULAR\_CARDIOMYOPATHY\_ARVC      
 Blue-Pink O' Gram in the Space of the Analyzed GeneSet

  

Fig 3: KEGG\_ARRHYTHMOGENIC\_RIGHT\_VENTRICULAR\_CARDIOMYOPATHY\_ARVC: Random ES distribution      
 Gene set null distribution of ES for **KEGG\_ARRHYTHMOGENIC\_RIGHT\_VENTRICULAR\_CARDIOMYOPATHY\_ARVC**
